# Long Non-Coding RNAs Guide the Fine-Tuning of Gene Regulation in B-Cell Development and Malignancy

**DOI:** 10.3390/ijms19092475

**Published:** 2018-08-21

**Authors:** Mette Dahl, Lasse Sommer Kristensen, Kirsten Grønbæk

**Affiliations:** 1 Department of Hematology, Rigshospitalet, Copenhagen University Hospital, DK-2100 Copenhagen, Denmark; mette.dahl.01@regionh.dk; 2 Biotech Research and Innovation Centre, BRIC, Copenhagen University, DK-2100 Copenhagen, Denmark; 3 Department of Molecular Biology and Genetics (MBG), Aarhus University, DK-8000 Aarhus, Denmark; lasse@mbg.au.dk; 4 Interdisciplinary Nanoscience Center (iNANO), Aarhus University, DK-8000 Aarhus, Denmark

**Keywords:** long non-coding RNA, circular RNA, B-cell development, mantle cell lymphoma (MCL), acute lymphoblastic leukemia (ALL), chronic lymphocytic leukemia (CLL), diffuse large B-cell lymphoma (DLBCL), burkitt lymphoma (BL), multiple myeloma (MM), gene regulation

## Abstract

With the introduction of next generation sequencing methods, such as RNA sequencing, it has become apparent that alterations in the non-coding regions of our genome are important in the development of cancer. Particularly interesting is the class of long non-coding RNAs (lncRNAs), including the recently described subclass of circular RNAs (circRNAs), which display tissue- and cell-type specific expression patterns and exert diverse regulatory functions in the cells. B-cells undergo complex and tightly regulated processes in order to develop from antigen naïve cells residing in the bone marrow to the highly diverse and competent effector cells circulating in peripheral blood. These processes include V(D)J recombination, rapid proliferation, somatic hypermutation and clonal selection, posing a risk of malignant transformation at each step. The aim of this review is to provide insight into how lncRNAs including circRNAs, participate in normal B-cell differentiation, and how deregulation of these molecules is involved in the development of B-cell malignancies. We describe the prognostic value and functional significance of specific deregulated lncRNAs in diseases such as acute lymphoblastic leukemia, chronic lymphocytic leukemia, mantle cell lymphoma, diffuse large B-cell lymphoma, follicular lymphoma, Burkitt lymphoma and multiple myeloma, and we provide an overview of the current knowledge on the role of circRNAs in these diseases.

## 1. Introduction

**Figure 1:**
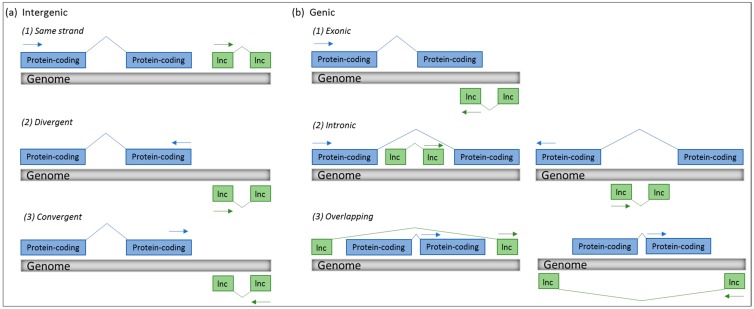
Positional classification of long non-coding RNAs (lnc) according to the GENCODE v7 catalogue of human long non-coding RNAs. (**a**) Intergenic long non-coding RNAs (lncRNAs) are located in between two independent genes and can be transcribed either from the same strand (1) or antisense in a divergent (2) or convergent (3) manner. (**b**) Genic lncRNAs are subdivided into: (1) exonic lncRNAs that intersect a protein-coding gene by at least 1 bp, (2) intronic lncRNAs that reside within the intron of a protein coding gene as either sense or antisense, and (3) overlapping lncRNAs that contain a protein-coding gene within an intron, as either sense or antisense. All antisense transcripts can be transcribed in a head-to head manner, as shown in (2), or in a tail-to-tail manner, as shown in (1). Arrows indicate direction of transcription of the protein-coding gene (blue) or the lncRNA (green). A final category is termed “processed transcript”, and this is used when the locus does not contain an open reading frame, but it does not fall into any of the other categories.

Long non-coding RNA (lncRNA) comprises a large and heterogeneous class of transcripts, arbitrarily defined as being more than 200 nucleotides in length, and generally characterised by low sequence conservation. However, some lncRNAs are evolutionary conserved with preserved functions, and the fact that negative selection acts on the promoters of these transcripts underlines the fact that they are strictly regulated and functionally important [1]. In humans, more than 20,000 protein-coding genes account for less than 3% of the entire genome, while approximately 80% has been shown to be non-coding, but functional [2]. According to the latest GENCODE update, 15,778 lncRNA transcripts have been annotated [3], and expression of lncRNAs, that are mainly localised in the nucleus, are generally lower and more tissue-specific than messenger RNAs (mRNAs) [4]. For a positional categorisation based on the GENCODE catalogue of lncRNAs, see Figure 1 [4,5]. Note that lncRNA terminology is often conflicting and overlapping, and currently, an unambiguous system for annotation of lncRNAs does not exist. For further insights into these difficulties of lncRNA classification, annotation, and terminology, we refer to Wright et al. and Laurent et al. [6,7].

**Figure 2:**
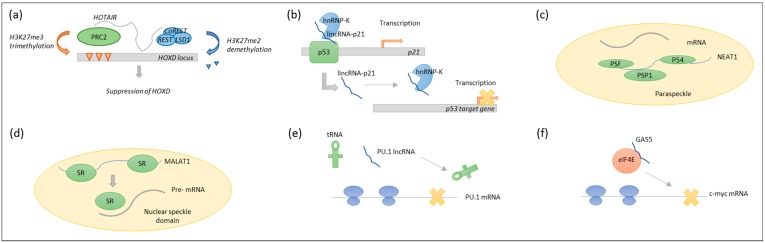
Cellular functions of lncRNAs. (**a**) LncRNAs as scaffolds for histone modification enzymes. *Homeobox transcript antisense intergenic RNA* (*HOTAIR*) tethers both polycomb repressive complex 2 (PRC2) and coREST/REST/LSD1, thereby specifying the pattern of histone modification on target genes. (**b**) lncRNAs can regulate gene expression in *cis* or in *trans*. Shown here is *large intergenic non-coding RNA p21 (lincRNA-p21)*, which act in *cis* as a coactivator for p53-dependent transcription of p21, or in *trans* by interacting with heterogenous nuclear ribonucleoprotein K (hnRNP-K) to mediate repression of distant p53 target genes (**c**) LncRNAs such as *nuclear enriched abundant transcript 1 (NEAT1)* can retain mRNAs in the nucleus by associating with paraspeckle proteins such as PSF, PSP1, and p54. (**d**) In tissue-specific alternative splicing, lncRNAs participate by recruiting serine/arginine splicing factors (SR) to nuclear speckles, and thereby to the target pre-mRNAs, as shown for *metastasis associated lung adenocarcinoma transcript 1 (MALAT1)*. (**e**) LncRNAs can serve as decoys inhibiting protein synthesis, here exemplified by antisense lncRNA *PU.1*, which blocks transfer RNA (tRNA) recruitment by inhibiting elongation through translation elongation factor eEF1a1, thereby inhibiting hematopoietic transcription factor PU.1 mRNA translation. (**f**) LncRNA *growth-specific 5 (GAS5)* interacts with translation initiation factor eIF4E to suppress the translation of c-myc mRNA.

LncRNAs exert diverse functions such as chromatin remodelling, transcriptional regulation and posttranscriptional processing [8,9,10]. For instance, *homeobox transcript antisense intergenic RNA (HOTAIR)* regulate gene expression by serving as a scaffold for histone modification enzymes [11], and *large intergenic non-coding RNA p21 (lincRNA-p21)* can serve as a transcriptional coactivator or repressor [12,13]. *Nuclear enriched abundant transcript 1 (NEAT1)* participates in nuclear retention of mRNAs [14], and *metastasis associated lung adenocarcinoma transcript 1 (MALAT1)* is involved in alternative splicing [15]. In the cytoplasm, lncRNAs can act as decoys, inhibiting protein synthesis of host genes [16], or regulating the translation of specific transcripts, which has been shown for *growth-specific 5 (GAS5)* [17]. See Figure 2.

A newly recognised subclass of lncRNA, named circular RNA (circRNA), have emerged as important gene regulatory molecules. CircRNAs are formed through a backsplicing event, which covalently link the 3′ end of an exon to the 5′ end of the same or an upstream exon. Most circRNAs originate from a host gene, and their biogenesis is facilitated either by flanking homologous inverted repeats bringing the splice sites in close proximity, or by dimerization of RNA binding proteins [18,19].

**Figure 3:**
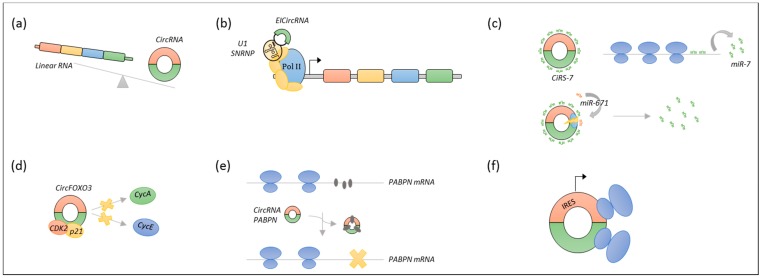
Proposed functions of circular RNAs (circRNAs). (**a**) CircRNAs can regulate gene expression indirectly through competition with canonical splicing. (**b**) Exon-intron circular RNAs (EICircRNAs) can directly enhance the transcription of host genes through interaction with the transcription complex. (**c**) CircRNAs can function as microRNA (miRNA) sponges, here exemplified by circular sponge for miR-7 (ciRS-7), which has >70 binding sites for miR-7. In the presence of ciRS-7, miR-7 target mRNA will be released from the miRNA-mediated repression. Upon binding of miR-671, an argonaute 2 (AGO2)-mediated cleavage occurs, providing immediate activation of miR-7 (**d**) CircRNAs can function as protein scaffolds or decoys. CircFOXO3 forms a ternary complex with p21 and cyclin-dependent kinase 2 (CDK2), blocking the interaction with cyclin A and cyclin E, thereby retarding cell cycle entry. (**e**) CircRNAs can serve as specific or global regulators of protein translation. CircRNA polyadenylate-binding protein nuclear (PABPN) sequesters the RNA-binding protein Hu Antigen R (HuR), leading to decreased PABPN mRNA translation. (**f**) Under certain circumstances, circRNAs have been reported to be translated.

These molecules also display tissue- and disease-specific expression patterns, but, unlike other lncRNAs, many circRNAs are highly evolutionary conserved [18,20]. Due to the lack of free ends, circRNAs are highly stable molecules that are resistant to exonucleases [18], and thus they hold great potential as diagnostic and prognostic biomarkers. It has been shown that particular circRNAs function as direct or indirect regulators of host gene expression at the transcriptional level [21,22], as sponges of microRNAs (miRNAs) [23,24], as protein scaffolds [25], or as specific or global regulators of protein translation [26,27]. Recent studies have reported that some circRNAs under certain circumstances can serve as templates for translation [28,29,30,31], yet the vast majority of circRNAs are considered to be non-coding [32]. See Figure 3.

Several studies have shown that lncRNAs and circRNAs are involved in cell differentiation and tissue development [33,34,35,36], and they are central players in the pathogenesis of various diseases including cancer [37,38,39,40]. However, regarding B-cell malignancies, only a limited number of studies have examined the role of lncRNAs and circRNAs as drivers of carcinogenesis, and assessed whether strict regulation of these molecules is necessary for normal B-cell differentiation. For a comprehensive review on B-cell development and a description of how different subtypes of lymphoma are proposed to arise from different stages of B-cell maturation, we refer to Küppers et al. [41].

In this review, we provide an overview of the current studies examining the expression and functions of lncRNAs and circRNAs in B-cell development and oncogenic transformation into various B-cell malignancies.

## 2. LncRNA in B-Cell Development

LncRNA expression profiling during B-cell development has been performed in several studies that report cell-type specific expression patterns at various stages of B-cell development [42,43,44,45,46,47]. Specifically, one study reported expression of antisense lncRNAs such as MYB-AS1, SMAS-AS1, and LEF-AS1 originating from protein-coding genes with known functions in B-cell development, and a lincRNA named CTC-436K13.6, in early B-cell subsets [44].

Furthermore, the cells in proliferative stages of B-cell development, including both precursor B-cells and centroblasts in the germinal centres (GC), showed co-expression of mitotic cell cycle genes with several lncRNAs including the bidirectional lncRNA named colorectal neoplasia differentially expressed (CRNDE) [44]. Tayari et al. only examined mature B-cell populations and reported similar expression profiles of lncRNAs in naïve and memory B-cell subsets, but significant differential expression in the cells of the highly proliferative GC [46], a pattern that was also observed in two other profiling studies [43,47], suggesting that lncRNAs might play a pivotal role here. A study in mice reported that expression of paired box 5 (PAX5), a transcription factor that is crucial for B-cell commitment [48], led to differential expression of several lncRNAs, including enhancer-associated lncRNAs, which were shown to be bound by PAX5, and for which human orthologs have been described [47]. Another study in mice proposed a dominant role of germ-line transcribed lncRNAs during V(D)J recombination in progenitor B-cells [49]. The most abundant transcripts were the PAX5-activated intergenic repeat (PAIR) elements *PAIR4* and *PAIR6*, which are transcribed antisense to *PAX5*. The authors propose that these lncRNAs are essential for locus compaction, positioning neighbouring heavy chain genes optimally for gene rearrangements to occur [49]. Remarkably, B-cells that are deficient of the transcription factor YY1, which is necessary for distal V_H_ gene rearrangements and precursor B-cell transition [50], displayed a marked reduction in both antisense transcription and DNA looping between the PAIR promoter and the intronic enhancer, compared to B-cells with intact YY1 [49], supporting a pivotal role of *PAIR4* and *PAIR6* in V(D)J recombination during B-cell development. YY1 has also been proposed to interact with and relocate the lncRNA *Xist*, to the inactivated X-chromosome in activated B-cells, thereby changing the X-linked gene regulation in these cells compared to antigen naïve B-cells [51].

Finally, high levels of the protein-coding PU.1 result in macrophage development, while low levels lead to B-cell commitment [52]. In mice, high PU.1 expression levels are necessary for the transition of B1 to B2 cells, and since failure to perform this lineage commitment has been linked to malignant transformation [53], the antisense *PU.1* could be a driver of lymphomagenesis by inhibiting the expression of PU.1 at the translational level [16].

## 3. Long Non-Coding RNA Expression in Various B-Cell Malignancies

With the development and wide accessibility of high-throughput technologies such as RNA sequencing (RNA-seq) [54], it has become evident that deregulation in the non-coding regions of our genome play a pivotal role in oncogenic transformation [39]. Here, we provide an overview of the current knowledge on expression and function of lncRNAs in B-cell malignancies, based on disease entities. Table 1 lists the lncRNA candidates, which have been examined by more than one study, and provide an overview of the functional characterisation and prognostic value of these lncRNAs across various B-cell malignancies.

### 3.1. Acute Lymphoblastic Leukemia

In B-cell acute lymphoblastic leukemia (B-ALL), a specific lncRNA expression pattern was observed in patients with *MLL*-rearranged B-ALL, compared to normal controls and to B-ALL patients without rearrangements [55]. The *MLL* gene is associated with adverse outcome [56], and the lncRNA expression pattern could stratify patients based on the *MLL* fusion partner. In a larger study, a lncRNA signature could predict whether patients carried the cytogenetic subtype *EVT6-RUNX1, TCF3-PBX1* or *MLL* rearrangements, and these transcripts were therefore termed B-ALL-associated long non-coding RNAs (BALR). The most differentially expressed lncRNAs between leukemic and normal B-cells and within the cytogenetic subtypes were *BALR-1*, *BALR-2*, *BALR-6*, and *LINC00958* [57], and interestingly, both *BALR-2* [57] and *BALR-6* [58] knockdown increased apoptosis. The expression of the lincRNA *CASC15*, which is located adjacent to the transcriptional activator *SOX4*, was significantly higher expressed in patients with *EVT6-RUNX1* and *TCF3-PBX1* translocations than in patients with *MLL* rearrangements [57]. Expression of *CASC15* and *SOX4* was positively correlated, and functional studies suggested that *CASC15* could enhance the transcription of *SOX4* through YY1 [59]. Furthermore, the expression of four lncRNAs, *lnc-NKX2-3-1*, *lnc-RTN4R*, *lnc-TIMM21-5*, and *lnc-ASTN1-1*, was shown to be specifically regulated by the oncogenic fusion protein *EVT6/RUNX1* [60], which is known to be associated with favourable prognosis [61]. In order to explore previously unannotated transcripts, one study used RNA-seq and identified 799 lncRNAs deregulated in B-ALL compared to controls [62].

These lncRNAs were more B-ALL-subtype specific than protein-coding genes and of note, this study confirmed the study by Fernando et al. [57], showing upregulation of *BALR-1* and *LINC00958* and increased *BALR-2* expression in patients with *EVT6/RUNX1* and *MLL* rearrangements, respectively. It was also shown that one of the upregulated lncRNAs, *RP11-137H2.4* [62], reduced cell-proliferation, increased apoptosis upon cytotoxic treatment, and partly restored sensitivity to prednisolone in resistant cell lines [86].

Finally, the methylation patterns of CpG islands in antisense lncRNA coding regions have been examined, showing significant hypermethylation within the gene bodies of antisense lncRNAs in ALL compared to progenitor B-cells, yet expression levels of these lncRNAs were not examined [87]. This hypermethylation was also observed in naïve B-cells and precursor B-cells, questioning whether the differential methylation patterns were disease-specific or just related to the maturation states of the B-cells.

### 3.2. Chronic Lymphocytic Leukemia 

The expression of several lncRNAs has been shown to be regulated by *TP5*3 [88], the deletions of which are associated with adverse outcome in patients with chronic lymphocytic leukemia (CLL) [89]. Recently, a study showed that *lincRNA-p21* is upregulated upon irradiation in cultured primary CLL cells with wild type *TP53*, leading to decreased cell viability, a mechanism lacking in cells with *TP53* mutations or deletions [67]. Interestingly, the level of cell-free circulating *lincRNA-p21* has been shown to be significantly lower in CLL patients compared to controls, suggestive of tumour-suppressive functions of *lincRNA-p21* [68].

Myocardial infarction-associated transcript (*MIAT*), a lncRNA involved in RNA splicing [71], has been correlated with adverse outcome in CLL [70]. Two lncRNAs, *DLEU1* and variant *DLEU2*, originate from the chromosomal region 13q14.3, which is frequently deleted in CLL, an alteration that is associated with adverse outcome [90]. *DLEU1* and variant *DLEU2* are hypomethylated at the 5’-ends, and upregulated in CLL cells compared to B-cells from controls. On the contrary, the protein-coding genes, including the *DLEU2* region containing the *miR15a/16.1* cluster, were downregulated in CLL cells. The inverse relationship of this gene cluster with *DLEU1* and variant *DLEU2* suggests that they act in *cis* and take part in a multi-regulatory network of protein-coding genes, lncRNAs, and microRNAs (miRNAs) that might be involved in CLL pathogenesis [91].

DNA methylation levels of the promoter regions of two lncRNAs, *AC012065.7*, and *CRNDE*, have been shown to be inversely correlated with their expression levels. Compared to normal controls, CLL samples displayed higher methylation levels of the *CRNDE* promoter, and lower methylation levels of the *AC012065.7* promoter, both associated with poor overall survival (OS) [73]. Moreover, expression of *AC012065.7* and *CRNDE* were positively correlated with expression of the protein-coding genes *GDF7* and *IRX5* respectively; both are encoded close to the lncRNAs, suggesting *cis*-regulation.

Epigenetic silencing was also shown for the lncRNA named *BM742401*, which is embedded in a CpG island. The promoter of this lncRNA was methylated in CLL cell lines, and unmethylated in bone-marrow samples from normal controls. Treatment with the hypomethylating agent 5-Aza-2’-deoxycytidine led to an increase in expression levels of *BM742401*, and overexpression in CLL cell lines reduced cellular proliferation and enhanced the intrinsic apoptotic pathway. However, in diagnostic CLL bone marrow samples, the methylation status of *BM742401* was not correlated with disease stage or OS [92].

Ronchetti et al. identified a lncRNA classifier consisting of 24 differentially expressed lncRNAs that could accurately discriminate CLL patients in early stage (Binet A) from normal controls [93]. Furthermore, it was found that expression levels of two of these lncRNAs, *lnc-IRF2-3* and *lnc-KIAA17554*, were significantly associated with progression-free survival (PFS) independent of common risk factors such as *NOTCH* and *IGHV* mutational status, CD38 and ZAP70 expression levels, and chromosomal aberrations.

Another large RNA-seq experiment showed differential expression of 127 lncRNAs and 61 pseudogenes in CLL compared to controls. The pseudogenes *CD24P4* and *PSMD10P1*, which have corresponding protein-coding genes involved in B-cell activation and oncogenesis, respectively, were both upregulated; however, the functions of these molecules remain to be examined [94].

A microarray study identified eight lncRNAs differentially expressed in CLL patients compared to controls. One of these, *translation regulatory long non-coding RNA1 (treRNA1)* was significantly higher expressed in unmutated *IGHV* samples [95], and served as an independent prognostic marker for shorter PFS in patients receiving fludarabine and cyclophosphamide. Overexpression of *treRNA1* in a CLL cell line led to reduced cell death, suggesting that *treRNA1* decreases DNA damage and sensitivity to chemotherapy, thereby explaining the shorter PFS. Recently, high expression of an antisense lncRNA from *ARHGAP15* termed *AC092652.2-202* was shown to be associated with shorter time to treatment in CLL patients. This effect was independent of *IGHV* mutational status and disease stage, and gene set enrichment analysis showed that genes potentially modulated by this lncRNA were significantly enriched in the NFκB, apoptosis, and p53 pathways [96].

### 3.3. Mantle Cell Lymphoma

*MALAT1* is upregulated in tumour tissues from mantle cell lymphoma (MCL) patients compared to normal B-cells, and knockdown of MALAT1 results in cell cycle arrest due to upregulation of p21 and p27 through enhancer of zeste homolog 2 (EZH2), a component of the polycomb repressive complex 2 (PRC2) [63]. The non-selective pan-histone deacetylase inhibitor Panobinostat [97] and the global histone methylation inhibitor 3-deazanoplanocin A (DzNep) [98] act synergistically to deplete EZH2 and induce apoptosis in primary MCL cells in vitro [99]. Yet, only Panobinostat has been investigated and it showed activity in patients with MCL; unfortunately however, the treatment led to severe thrombocytopenia [100], and it is intriguing whether *MALAT1* could serve as an alternative therapeutic target. EZH2, which is upregulated in MCL [101], and linked to adverse outcome [102], has been shown to bind a lncRNA termed *ROR1-AS1*, which increases cell proliferation in MCL cell lines; however, *ROR1-AS1* expression was not significantly different when comparing MCL patients to normal controls [103]. The promoter region of *FAS-AS1* is regulated by EZH2 as well, and this lncRNA serves to modulate alternative splicing of the *FAS* gene, a central molecule in the extrinsic apoptosis pathway. *FAS-AS1* sequesters RBM5, leading to decreased exon skipping and upregulation of the membrane-bound isoform, whereas the soluble isoform (sFAS) that inhibits apoptosis, is downregulated [104]. Treatment with DzNep or Ibrutinib, which targets BTK, increased FAS ligand-mediated apoptosis in lymphoma cell lines by abolishing the EZH2-mediated repression of *FAS-AS1* expression, leading to decreased expression of sFAS.

It is well established that *MALAT1* has oncogenic functions in various cancer types [105], and depletion of *MALAT1* leads to *TP53* upregulation, possibly due to double-stranded DNA damage [106], indicating that *TP53* might be a target of *MALAT1* as well. Finally, knockdown of the proposed tumour-suppressor *GAS5* resulted in decreased levels of apoptosis in MCL cell lines, and a significant decrease in the treatment effect of mTOR inhibitors [81].

### 3.4. Diffuse Large B-Cell Lymphoma and Follicular Lymphoma

One study examined RNA-seq data sets of diffuse large B-cell lymphoma (DLBCL) and showed that normal tissue displayed the highest average number of lncRNAs per sample, followed by tumour samples and cell lines, suggesting that expression of lncRNAs might be negatively correlated to the proliferation states of the cells [107]. In total, 2632 novel lncRNAs were identified in this study, most of which were only expressed in malignant cells.

Using microarrays, another study identified 1648 upregulated and 2671 downregulated lncRNAs in germinal centre B-cell (GCB)-DLBCL cell lines compared to normal B-cells [76]. The expression patterns of five of these lncRNAs (*AFAP-AS1*, *OR2A1-AS1*, *NAALADL2-AS2*, *HOTAIRM1*, and *RP4-545C24.1*.) were confirmed in clinical samples from GCB-DLBCL patients and lymph nodes from normal controls by RT-qPCR. A similar approach was used to study differentially expressed lncRNAs between normal B-cells and DLBCL cell lines of both activated B-cells (ABC- and GCB-type) [77]. Interestingly, one of the candidates from the other study, *NAALADL2-AS2*, was also among the most upregulated lncRNAs in this study; however, no functional studies were performed.

By analysing microarray data from the gene expression omnibus (GEO) database, including more than 1000 DLBCL patients, a lncRNA signature based on expression of six lncRNAs, *SACS-AS1*, *MME-AS1*, *CSMD2-AS1*, *RP11-360F5.1*, *RP1125K19.1*, and *CTC-467M3.1*, was significantly correlated with OS in two independent patient cohorts [108]. Interestingly, the signature could improve risk stratification by predicting the survival of patients with identical international prognostic index (IPI) scores. In another study, the same authors reanalysed the data sets and reported a novel signature consisting of 17 lncRNAs, which could, not only predict OS and PFS, but also distinguish ABC and GCB subtypes of DLBCL with more than 90% accuracy [109].

It has also been suggested that lncRNAs may play a crucial role in the chromosome breaks involved in typical gene rearrangements in hematologic malignancies. For example, the boundaries of the antisense lncRNA *RP11-211G3.3.1-1* from the *BCL6* locus, precisely match the boundaries of the *BCL6* translocation zone [110], and future studies should assess whether knockdown of this lncRNA could prevent *BCL6* translocation and potentially preclude lymphoma development.

Several studies have examined the prognostic significance of single lncRNAs in DLBCL. A promising candidate, *HOTAIR*, repress target genes through PRC2 [80], and two studies reported diverging results regarding the association of *HOTAIR* expression with OS [78,79]. Another study found that *P21-associated non-coding RNA (ncRNA) DNA damage activated (PANDA)* was downregulated in DLBCL samples compared to normal controls, and low expression was associated with poor OS. Mechanistically, *PANDA* was shown to be activated by p53, and downregulation of *PANDA* increased cell viability in DLBCL cells, whereas overexpression had the opposite effect [111]. *MALAT1* has also been proposed to play a role in DLBCL oncogenesis [64], yet expression levels have not been analysed in DLBCL patients. High expression of a lncRNA originating as a processed transcript from *paternally expressed 10 (PEG10)* has also been associated with decreased OS in DLBCL, and knockdown was shown to induce apoptosis in a DLBCL cell line [112]. Another lncRNA, *NONHSAG026900* was significantly lower expressed in DLBCL compared to normal B-cells in two independent patient cohorts, and expression levels could add predictive power to the IPI score, yet it was inferior to IPI when used as an independent prognostic marker [113].

In follicular lymphoma (FL), only one small study has examined lncRNA expression, and showed that 189 lncRNAswere aberrantly expressed between three patients with grade 3a FL and normal controls. Four candidates were validated with RT-qPCR, *and RP11-625 L16.3 and CTC-546 K23.1* were significantly upregulated, whereas *AP005530.2* and *AP005530.2* were significantly downregulated [114].

### 3.5. Burkitt’s Lymphoma

In Burkitt’s lymphoma (BL), translocations involving the proto-oncogene *MYC* and one of the three immunoglobulin loci are considered a pathogenic hallmark [115,116]. A comprehensive study analysed RNA-seq data and showed that a lncRNA that was transcribed from chromosome 8 was significantly correlated with MYC expression, and the authors termed it *MYC-induced long non-coding RNA (MINCR)*. The association of MYC and MINCR seemed to represent a general oncogenic mechanism, since it could also be detected in DLBCL, FL, CLL, and non-haematological cancers [117]. Upon *MINCR* knockdown, expression of cell-cycle related genes such as *AURKA*, *AURKB*, and *CDT1* was affected, leading to the perturbation of cell-cycle progression. These genes all displayed decreased MYC-binding in their promoter regions upon *MINCR* knockdown, suggesting that this lncRNA was involved in regulating MYC target genes. Furthermore, it has been proposed that MYC enhances rather that induces transcription of lncRNAs, since data from a *MYC*-repressible cell line expressed the same lncRNAs in the *MYC*-OFF state and the *MYC*-ON state, but at significantly different levels. The same study examined patient samples and identified a total of 974 differentially expressed lncRNAs when comparing nine CLL patients and 13 patients with BL, characterised by low and high MYC expression, respectively [118].

### 3.6. Multiple Myeloma

Compared to other B-cell malignancies, there are numerous studies on lncRNA expression in multiple myeloma (MM), including lncRNA profiling by both microarray [65,119,120,121,122] and RNA-seq [72,123]. One of these studies compared normal plasma cells to plasma cells from patients with monoclonal gammopathy of undetermined significance (MGUS), smoldering multiple myeloma (SMM), MM and plasma cell leukemia (PCL), and identified 160 differentially expressed lncRNAs. These included six lncRNAs that were validated by RT-qPCR, *MALAT1*, *GAS5*, *DLEU2*, *lnc-LRRC47-1*, *lnc-ANGPTL1-3*, and *lnc-SENP5-4*, the latter three being significantly deregulated in more aggressive diseases [65].

Interestingly, one of the RNA-seq studies reported that in 30 MM samples, 12 lncRNAs including *NEAT1*, *MALAT1*, *MIAT*, and *taurine upregulated 1 (TUG1)* were highly expressed, accounting for 64% of the reads mapping to lncRNAs [72]. The other RNA-seq study focused specifically on lincRNAs and determined a risk score based on a lincRNA signature consisting of the 14 lincRNAs with highest impact on PFS. The signature was validated in an independent cohort and could separate patients with high and low risk disease, with respect to both PFS and OS [123].

While many specific lncRNAs are proposed to have prognostic relevance in MM [120,122], even studies examining the same data sets identify different lncRNAs with prognostic potentials [120,121], and e.g. *MALAT1* has been proposed as a prognostic biomarker, yet studies report diverging results [66,68], emphasising the need for validation studies.

*Maternally expressed 3 (MEG3)*, an imprinted lncRNA transcribed antisense to *BMP4*, was shown to promote BMP4-induced osteogenic differentiation of mesenchymal stem cells isolated from bone marrow samples. The level of *MEG3* was significantly lower in MM patients compared to controls [124], which was suggested to be due to promoter hypermethylation [125]. Promoter hypermethylation has also been proposed to cause downregulation of *KIAA0495 (TP73-AS1)* [126] in MGUS and MM patients, but not in healthy controls [127]. A different antisense lncRNA from the tumour suppressor gene *TP73,* has been reported to be downregulated in MM as well [65].

Polymorphisms may also influence the expression of lncRNAs, as is the case for *antisense non-coding RNA in the INK4-ARF locus (ANRIL)*, which is highly expressed in individuals carrying TT as compared to CC or CT at the *rs2151280* polymorphism. High *ANRIL* expression results in lower expression of p15, p14ARF, and p16, possibly explaining why this polymorphism is significantly associated with poor PFS in MM [128].

Studies have also focused on how lncRNAs interact with miRNAs and influence cellular processes via downstream signalling pathways. For instance, *OIP5-AS1* downregulation has been shown to result in accumulation of miR-410, which target KLF-10, leading to increased cell cycle progression, proliferation, and inhibition of apoptosis through the PTEN/PI3K/AKT pathway [129]. Similarly, *FEZF1-AS1* act as a competing endogenous RNA (ceRNA) for miR-610, thereby releasing the miR-610-mediated inhibition of AKT3 [130]. *Colon cancer-associated transcript 1 (CCAT1)* was also shown to act as a ceRNA by binding miR-181a-5p, thereby releasing inhibition of *HOXA1* expression. *CCAT1* expression was significantly higher in MM patients compared to controls, and knockdown resulted in suppression of MM tumour growth in MM cell lines and mice [131].

The role of lncRNA in drug response has been investigated as well. Six upregulated and nine downregulated lncRNAs were identified in both proteasome-inhibitor-resistant MM cell lines and isolated CD138 cells from MM patients compared to proteasome-inhibitor-sensitive cells or CD138 cells from healthy controls [132].

STAT3, a transcription factor linked to MM oncogenesis [133], has been shown to induce specific lncRNAs, termed STAT3-induced lncRNAs (STAiRs), upon activation of IL-6. STAiRs included both nuclear-retained lncRNAs that inhibit tumour-suppressive functions specific for MM, and lncRNAs that were ubiquitously expressed in various tumours and seemed to be involved in chromatin silencing [134].

Other lncRNAs suggested to play a role in MM include *NEAT1* [69], *MIAT* [72], *CRNDE* [75], *urothelial cancer associated 1 (UCA1)* [82,83], *H19* [84,85], *protein disulfide isomerase family a member 3 pseudogene 1 (PDIA3P)*, and *prostate cancer associated transcript 1 (PCAT1)* [135].

## 4. Circular RNA in B-Cell Development and Malignancies

Studies examining the role of circRNAs in the pathogenesis of B-cell malignancies are very sparse. The circRNA research field is quite new; however, increasing scientific interest has emerged since the discovery that ciRS-7 contains approximately 70 binding sites for the proposed tumour-suppressor miR-7 [23,24], indicating that ciRS-7 could have a central role in tumour development [136]. However, compared to lncRNAs, circRNAs are more difficult to study, primarily because they lack poly(A) tails, and they are therefore discarded during library preparation for RNA-seq when using protocols that rely on a poly(A) purification step for removal of ribosomal RNA (rRNA). Thus, most publicly available RNA-seq data sets cannot be analysed for circRNA expression, and specific bioinformatic pipelines recognising the specific backsplicing junction of circRNAs need to be utilised [137]. Furthermore, substantial methodological challenges like template switching and rolling circle amplification during RT and amplification bias during PCR have been observed in the detection of circRNAs, and risk hampering the results [38,138,139].

One of the first studies to profile circRNA expression in both normal and malignant tissues reported that >700 circRNA candidates were identified in five samples from children with hyperdiploid B-ALL [20]. Host genes for the most highly expressed circRNAs included *ESYT2*, *FBXW4*, *CAMSAP1*, *KIAA0368*, *CLNS1A*, *FAM120A*, *MAP3K1*, *ZKSCAN1*, *MANBA*, *ZBTB46*, *NUP54*, *RARS*, and *MGA*, and all were confirmed by RT-qPCR using divergent primer design. In normal CD19, positive naïve B-cells, and CD34 positive hematopoietic stem cells, novel circRNA candidates were identified as well [20].

No studies have yet examined the role of circRNAs during normal B-cell development and differentiation; however, specific circRNA signatures characteristic for B-cells compared to T-cells and progenitors have been described [140]. Intriguingly, studies in mice have also revealed that a circRNA originating from *the D430042O09Rik* gene was constitutively expressed, and it was shown to bind cyclic GMP-AMP synthase (cGAS) to block its enzymatic activity, thereby protecting long-term hematopoietic stem cells (LT-HSCs) from cGAS-mediated IFN-I-driven exhaustion [141].

It has also been observed that chromosomal translocations can give rise to fusion-circRNAs, transcribed from exons of distinct genes [142]. For example, two circRNAs are transcribed from the *MLL-AF9* translocation observed in ALL, and both exert oncogenic properties; however, it remains to be determined whether chromosomal translocations characteristic for other B-cell malignancies also give rise to fusion-circRNAs. In BL, characterised by high MYC expression as described above, upregulation of the circRNAs ZDHHC11 and ZDNN11B was shown, along with upregulation of the MYC target MYB. These circRNAs contain multiple binding sites for miR-150, a miRNA that was downregulated in cell lines with high MYC expression. The authors proposed that ZDHHC11 and ZDNN11B act as ceRNAs that bind miR-150 in normal cells to inhibit proliferation, while in BL cells, the MYC-induced repression of miR-150 leads to increased proliferation through upregulation of ZDHHC11, ZDNN11B, and MYB [143]. Another circRNA that might be important in lymphomagenesis is circAmotl1, which has been shown to trigger tumourigenesis through nuclear translocation of MYC and upregulation of MYC targets [144]; however, no studies have yet examined whether circAmotl1 is an important oncogenic driver in B-cell malignancies with high MYC expression such as BL or DLBCL.

Finally, we have recently performed RNA-seq in MM and MCL cell lines to profile the landscape of circRNA expression in B-cell malignancies [145]. Several circRNAs, previously shown to be implicated in other cancers were identified, including ciRS-7 [136,146,147], circHIPK3 [148,149], circCCDC66 [150], circFBXW7 [31], circSMARCA5 [151,152], circCDYL [149], and circZKSCAN1 [153]. CircRNAs from host genes involved in lymphomagenesis and the development of MM were also detected, including *FOXP1* [154], *SETD3* [155], *EZH2* [156], *ATM* [157], *XPO1* [158], *CD11A (ITGAL)* [159], *WHSC1 (MMSET)* [160], and *IKZF3* [161]; the latter is not listed in circBase [162]. In this study, we also applied a new method for accurate quantification of circRNAs using the NanoString Technology [163]. This method is free of any enzymatic steps and is therefore less prone to the introduction of biases relating to RT and amplification steps, and because the technology is based on two short probes, it is well suited for examining RNA samples isolated from formalin-fixed paraffin-embedded (FFPE) tissues [164]. We demonstrated that high quality data on circRNA quantification, in RNA samples isolated from FFPE tissues of patients with various B-cell malignancies, could be obtained [145].

It is apparent that even though circRNAs are highly expressed in various B-cell malignancies, it remains to be elucidated whether some circRNAs are independent oncogenic drivers, and what their mechanisms of action are.

## 5. Conclusions

It is evident that lncRNAs play important roles in B-cell development and differentiation, and the tissue- and cell-type-specific distribution makes these molecules promising candidates as prognostic and diagnostic biomarkers. However, several obstacles preclude the characterisation and clinical use of these molecules. As briefly touched upon, methodological challenges like PCR amplification bias [165], and cross-hybridisation issues in microarray [166] can hamper results, and they may explain some of the divergence in the studies described above. Even though many lncRNA profiling studies have been carried out, very few lncRNAs with prognostic potential have been identified, and the findings have rarely been validated in independent patient cohorts.

Regarding characterisation of lncRNAs, the relatively low evolutionary conservation [167] limits the use of animal models to study their function. Even when performing in vitro loss-of-function studies using common techniques such as RNA interference (RNAi), numerous difficulties arise. The nuclear localisation of most lncRNAs makes RNAi less effective [168], and for lncRNAs expressed at high levels, complete loss-of-function can be hard to obtain [169]. A powerful tool for creating stable knockouts is the clustered regularly interspaced palindromic repeats (CRISPR) technique [170]. However, researchers have to be cautious when utilising this system for knockdown of lncRNAs, as it is difficult to avoid affecting the expression of protein-coding genes from the same locus [171]. The ability of lncRNAs and circRNAs to bind miRNAs has fostered the idea that these molecules are capable of regulating gene expression through the interaction with miRNAs. However, stoichiometric analyses of lncRNA:miRNA:mRNA ratios and circRNA:miRNA:mRNA ratios are rarely performed, and the ceRNA hypothesis is only supported for a few candidates with multiple binding sites for miRNAs in cells with high expression levels of the circRNA or lncRNA [172,173]. In spite of these challenges, great advancements within the field of lncRNA have been made, and novel techniques for functional characterisation and computational tools for studying the regulatory crosstalk between mRNAs, miRNAs, lncRNAs, and circRNAs are evolving [174,175].

When assessing the prognostic value of lncRNAs and circRNAs it is important that identified candidates are validated in independent patient cohorts. Preferably, RNA-seq should be performed to be able to identify previously unannotated transcripts, yet this is often limited by the poor quality of the RNA isolated from patient samples, particularly if these are conserved as FFPE tissue. Furthermore, one must bear in mind that novel lncRNA and circRNA candidates identified by RNA-seq need to be thoroughly validated, as this method is prone to the introduction of bias and artefacts through the RT and PCR amplification steps. Because of this, the NanoString technology, which is free of any enzymatic steps, holds great potential as a clinically applicable method for lncRNA [176] and circRNA [145] quantification.

In conclusion, lncRNAs, including circRNAs, play pivotal roles in B-cell development and oncogenic transformation, yet we are only beginning to understand the functions of these molecules and how they contribute in the fine-tuning of gene expression in normal and malignant tissues. Future studies should aim primarily at the functional characterisation of these molecules and to identify suitable biomarkers and therapeutically relevant targets.

## Figures and Tables

**Table 1:** LncRNAs with prognostic and/or functional impact in B-cell malignancies verified by more than one study.

Name	Samples *	Expression	Proposed Function	Prognostic Impact	Reference
*BALR-1*	118 B-ALL	↑	No functional studies	No association with PFS/OS	[57]
	56 B-ALL	↑	No functional studies	No association with PFS/OS	[62]
*BALR-2*	118 B-ALL, cell lines	↑	Promote cell survival via the inhibition of genes such as the proapoptotic *BIM* downstream of the glucocorticoid receptor	↑ in steroid resistant patients and patients with poor OS	[57]
	56 B-ALL	↑	No functional studies	No association with PFS/OS	[62]
*BALR-6*	118 B-ALL, cell lines, mice	↑	Promotes cell survival and inhibits apoptosis. Overexpression in mice leads to an increase in precursor cell populations	No association with PFS/OS	[57,58]
*LINC00958*	118 B-ALL	↑	No functional studies	No association with PFS/OS	[57]
	56 B-ALL	↑	No functional studies	No association with PFS/OS	[62]
*MALAT1*	40 MCL, cell lines	↑	Binds to EZH2 and induces transcriptional repression of targets such as p21 and p27	High vs. low expression: HR = 3	[63]
	DLBCL cell lines, xenograft mice	↑	KD induces the expression of autophagy-related proteins, improving chemotherapy-sensitivity	Not assessed	[64]
	33 SMM, 170 MM, 36 PCL	↑	Associated with TP53-mediated DNA damage response	Not assessed	[65]
	36 MM	↑	No functional studies	Change in expression (diagnosis /treatment) associated with PFS	[66]
*lincRNA-p21*	73 primary CLL cells	↑ in *TP53*^wt^ compared to *TP53*^del/mut^	Decrease cell viability in a p53-dependent manner upon DNA damage	Not assessed	[67]
	68 CLL plasma samples	↓	p53 dependent *cis*-upregulation of p21, leading to cell cycle control through interaction with PRC2	Not assessed	[12,68]
*NEAT1*	73 primary CLL cells	↑ in *TP53*^wt^ compared to *TP53*^del/mut^	Nuclear retention of mRNAs with inverted repeats	Not assessed	[14,67]
	51 MM, cell lines	↑	Binds to miR-193a leading to MCL-1 upregulation and steroid resistance	↑ in patients with poor OS	[69]
*MIAT*	67 CLL, cell lines	↑ in patients with bad outcome	KD of *MIAT*, or its positive regulator *OCT4*, induces apoptosis	↑ in patients with poor OS	[70]
	30 MM	↓ in patients with t (11;14)	Involved in alternative splicing		[71,72]
*CRNDE*	70 CLL	↓	Interacts with PRC2 and CoREST to modulate transcriptional repression	Promoter methylation associated with poor OS	[73,74]
	77 MM, cell lines	↑	Binds to miR-451 to induce proliferation and inhibit apoptosis	↑ in patients with poor OS	[75]
*NAALADL2-AS2*	10 DLBCL, cell lines	↑	Involved in p53, NFκB, and JAK/STAT signalling pathways (Gene Ontology Analysis)	Not assessed	[76,77]
*HOTAIR*	50 DLBCL, cell lines	↑	Cell cycle progression and apoptosis inhibition through PI3K/AKT/NFκβ pathways	↑ in patients with poor OS. *HOTAIR* > median vs. < median: HR = 3.1	[78]
	164 DLBCL	Not specified	Recruits PRC2 and induce silencing of target genes through H3K27me3	↑ (higher than cancer-free tissue) in patients with favourable OS	[79,80]
*GAS5*	33 SMM, 170 MM, 36 PCL	↑ in patients with 1q gain lesions	No functional studies	Not assessed	[65]
	MCL cell lines		KD reduces apoptosis and decreases the effects of mTOR inhibitors on cell viability	Not assessed	[81]
	MCL cell lines		*GAS5* interact with c-myc mRNA to reduce translation	Not assessed	[17]
*UCA1*	84 MM	↓	No functional studies	High vs. low expression: HR = 2	[82]
	60 MM, cell lines	↑	Involved in cell proliferation and inhibition of apoptosis	Not assessed	[83]
*H19*	30 MM	↑	Induce proliferation through NFκβ pathway	H19 ↑ in patients with poor PFS	[84]
	80 MM	↑	No functional studies		[85]

* The number of patient samples included are shown and/or the species in which any functional studies were carried out. ↑ designates significantly elevated expression levels, while ↓ designates significantly decreased expression levels compared to normal controls unless otherwise specified. Not assessed denotes that the prognostic significance of the lncRNA was not assessed in the specific study. Abbreviations: B-ALL, B-cell acute lymphoblastic leukemia; MCL, mantle cell lymphoma; DLBCL, diffuse Large B-cell lymphoma; SMM, smoldering multiple myeloma; MM, multiple myeloma; PCL, plasma cell leukemia; CLL, chronic lymphocytic leukemia; KD, knockdown; PFS, progression-free Survival; OS, overall survival; HR, hazard Ratio.

## Abbreviations

ABC-DLBCL	Activated B-cell diffuse large B-cell lymphoma
ALL	Acute lymphoblastic leukemia
ANRIL	Antisense non-coding RNA in the *INK4-ARF* locus
B-ALL	B-Cell Acute lymphoblastic leukemia
BALR	B-ALL-associated long non-coding RNAs
BL	Burkitt Lymphoma
CCAT1	Colon cancer associated transcript 1
ceRNA	Competing endogenous RNA
cGAS	Cyclic GMP-AMP synthase
CRISPR	Clustered regularly interspaced palindromic repeats
CRNDE	Colorectal neoplasia differentially expressed
circRNA	Circular RNA
ciRS-7	Circular RNA sponge for miR-7
CLL	Chronic lymphocytic leukemia
DLBCL	Diffuse large B-cell lymphoma
DzNep	3-deazanoplanocin A
FFPE	Formalin-fixed paraffin-embedded
FL	Follicular lymphoma
GAS5	Growth specific 5
GC	Germinal center
GCB-DLBCL	Germinal center diffuse large B-cell lymphoma
GEO	Gene expression omnibus
HOTAIR	The HOX transcript antisense intergenic RNA
IPI	International prognostic index
KD	Knockdown
lincRNA-p21	Large intergenic non-coding RNA p21
lncRNA	Long non-coding RNA
LT-HSCs	Long term hematopoietic stem cells
MALAT1	Metastasis associated lung adenocarcinoma transcript 1
MCL	Mantle cell lymphoma
MIAT	Myocardial infarction associated transcript
MINCR	MYC-induced long non-coding RNA
miRNA	MicroRNA
MEG3	Maternally expressed 3
MGUS	Monoclonal gammopathy of undetermined significance
MM	Maternally expressed 3
NEAT1	Nuclear enriched abundant transcript 1
OS	Overall survival
PAIR	PAX5 activated intergenic repeat
PANDA	P21 associated ncRNA DNA damage activated
PCAT1	Prostate cancer associated transcript 1
PEG10	Paternally expressed 10
PFS	Progression-free survival
PCL	Plasma cell leukemia
PDIA3P	Protein disulfide isomerase family A member 3 pseudogene 1
PRC2	Polycomb repressive complex 2
RNAi	RNA interference
RNA-seq	RNA sequencing
rRNA	Ribosomal RNA
SMM	Smoldering multiple myeloma
STAiRs	STAT3-induced lncRNAs
treRNA1	Translation regulatory long non-coding RNA1
TUG1	Taurine upregulated 1
UCA1	Urothelial cancer associated 1

## References

[1] Guttman M., Amit I., Garber M., French C., Lin M.F., Feldser D., Huarte M., Zuk O., Carey B.W., Cassady J.P. (2009). Chromatin signature reveals over a thousand highly conserved large non-coding RNAs in mammals. Nature.

[2] The ENCODE Project Consortium (2012). An integrated encyclopedia of DNA elements in the human genome. Nature.

[3] Uszczynska-Ratajczak B., Lagarde J., Frankish A., Guigó R., Johnson R. (2018). Towards a complete map of the human long non-coding RNA transcriptome. Nat. Rev. Genet..

[4] Derrien T., Johnson R., Bussotti G., Tanzer A., Djebali S., Tilgner H., Guernec G., Martin D., Merkel A., Knowles D.G. (2012). The GENCODE v7 catalog of human long noncoding RNAs: Analysis of their gene structure, evolution, and expression. Genome Res..

[5] Harrow J., Frankish A., Gonzalez J.M., Tapanari E., Diekhans M., Kokocinski F., Aken B.L., Barrell D., Zadissa A., Searle S. (2012). GENCODE: The reference human genome annotation for The ENCODE Project. Genome Res..

[6] Wright M.W. (2014). A short guide to long non-coding RNA gene nomenclature. Hum. Genom..

[7] St. Laurent G., Wahlestedt C., Kapranov P. (2015). The Landscape of long noncoding RNA classification. Trends Genet..

[8] Mercer T.R., Dinger M.E., Mattick J.S. (2009). Long non-coding RNAs: Insights into functions. Nat. Rev. Genet..

[9] Cawley S., Bekiranov S., Ng H.H., Kapranov P., Sekinger E.A., Kampa D., Piccolboni A., Sementchenko V., Cheng J., Williams A.J. (2004). Unbiased Mapping of Transcription Factor Binding Sites along Human Chromosomes 21 and 22 Points to Widespread Regulation of Noncoding RNAs. Cell.

[10] Ponjavic J., Ponting C.P., Lunter G. (2007). Functionality or transcriptional noise? Evidence for selection within long noncoding RNAs. Genome Res..

[11] Tsai M.-C., Manor O., Wan Y., Mosammaparast N., Wang J.K., Lan F., Shi Y., Segal E., Chang H.Y. (2010). Long Noncoding RNA as Modular Scaffold of Histone Modification Complexes. Science.

[12] Dimitrova N., Zamudio J.R., Jong R.M., Soukup D., Resnick R., Sarma K., Ward A.J., Raj A., Lee J.T., Sharp P.A. (2014). LincRNA-p21 Activates p21 In *cis* to Promote Polycomb Target Gene Expression and to Enforce the G1/S Checkpoint. Mol. Cell.

[13] Huarte M., Guttman M., Feldser D., Garber M., Koziol M.J., Kenzelmann-Broz D., Khalil A.M., Zuk O., Amit I., Rabani M. (2010). A Large Intergenic Noncoding RNA Induced by p53 Mediates Global Gene Repression in the p53 Response. Cell.

[14] Clemson C.M., Hutchinson J.N., Sara S.A., Ensminger A.W., Fox A.H., Chess A., Lawrence J.B. (2009). An Architectural Role for a Nuclear Non-coding RNA: NEAT1 RNA is Essential for the Structure of Paraspeckles. Mol. Cell.

[15] Tripathi V., Ellis J.D., Shen Z., Song D.Y., Pan Q., Watt A.T., Freier S.M., Bennett C.F., Sharma A., Bubulya P.A. (2010). The Nuclear-Retained Noncoding RNA MALAT1 Regulates Alternative Splicing by Modulating SR Splicing Factor Phosphorylation. Mol. Cell.

[16] Ebralidze A.K., Guibal F.C., Steidl U., Zhang P., Lee S., Bartholdy B., Jorda M.A., Petkova V., Rosenbauer F., Huang G. (2008). PU.1 expression is modulated by the balance of functional sense and antisense RNAs regulated by a shared *cis*-regulatory element. Genes Dev..

[17] Hu G., Lou Z., Gupta M. (2014). The Long Non-Coding RNA GAS5 Cooperates with the Eukaryotic Translation Initiation Factor 4E to Regulate c-myc Translation. PLoS ONE.

[18] Jeck W.R., Sorrentino J.A., Wang K., Slevin M.K., Burd C.E., Liu J., Marzluff W.F., Sharpless N.E. (2013). Circular RNAs are abundant, conserved, and associated with ALU repeats. RNA.

[19] Conn S.J., Pillman K.A., Toubia J., Conn V.M., Salmanidis M., Phillips C.A., Roslan S., Schreiber A.W., Gregory P.A., Goodall G.J. (2015). The RNA binding protein quaking regulates formation of circRNAs. Cell.

[20] Salzman J., Gawad C., Wang P.L., Lacayo N., Brown P.O. (2012). Circular RNAs are the predominant transcript isoform from hundreds of human genes in diverse cell types. PLoS ONE.

[21] Ashwal-Fluss R., Meyer M., Pamudurti N.R., Ivanov A., Bartok O., Hanan M., Evantal N., Memczak S., Rajewsky N., Kadener S. (2014). CircRNA Biogenesis competes with Pre-mRNA splicing. Mol. Cell.

[22] Li Z., Huang C., Bao C., Chen L., Lin M., Wang X., Zhong G., Yu B., Hu W., Dai L. (2015). Exon-intron circular RNAs regulate transcription in the nucleus. Nat. Struct. Mol. Biol..

[23] Hansen T.B., Jensen T.I., Clausen B.H., Bramsen J.B., Finsen B., Damgaard C.K., Kjems J. (2013). Natural RNA circles function as efficient microRNA sponges. Nature.

[24] Memczak S., Jens M., Elefsinioti A., Torti F., Krueger J., Rybak A., Maier L., Mackowiak S.D., Gregersen L.H., Munschauer M. (2013). Circular RNAs are a large class of animal RNAs with regulatory potency. Nature.

[25] Du W.W., Yang W., Liu E., Yang Z., Dhaliwal P., Yang B.B. (2016). Foxo3 circular RNA retards cell cycle progression via forming ternary complexes with p21 and CDK2. Nucleic Acids Res..

[26] Holdt L.M., Stahringer A., Sass K., Pichler G., Kulak N.A., Wilfert W., Kohlmaier A., Herbst A., Northoff B.H., Nicolaou A. (2016). Circular non-coding RNA ANRIL modulates ribosomal RNA maturation and atherosclerosis in humans. Nat. Commun..

[27] Abdelmohsen K., Panda A.C., Munk R., Grammatikakis I., Dudekula D.B., De S., Kim J., Noh J.H., Kim K.M., Martindale J.L. (2017). Identification of HuR target circular RNAs uncovers suppression of PABPN1 translation by CircPABPN1. RNA Biol..

[28] Pamudurti N.R., Bartok O., Jens M., Ashwal-Fluss R., Stottmeister C., Ruhe L., Hanan M., Wyler E., Perez-Hernandez D., Ramberger E. (2017). Translation of CircRNAs. Mol. Cell.

[29] Legnini I., Di Timoteo G., Rossi F., Morlando M., Briganti F., Sthandier O., Fatica A., Santini T., Andronache A., Wade M. (2017). Circ-ZNF609 Is a Circular RNA that Can Be Translated and Functions in Myogenesis. Mol. Cell.

[30] Yang Y., Fan X., Mao M., Song X., Wu P., Zhang Y., Jin Y., Yang Y., Chen L., Wang Y. (2017). Extensive translation of circular RNAs driven by N6-methyladenosine. Cell Res..

[31] Yang Y., Gao X., Zhang M., Yan S., Sun C., Xiao F., Huang N., Yang X., Zhao K., Zhou H. (2018). Novel Role of FBXW7 Circular RNA in Repressing Glioma Tumorigenesis. J. Natl. Cancer Inst..

[32] Stagsted L.V.W., Nielsen K.M., Daugaard I., Hansen T.B. (2018). Non-coding AUG circRNAs constitute an abundant and conserved subclass of circles. bioRxiv.

[33] Kristensen L.S., Okholm T.L.H., Venø M.T., Kjems J. (2017). Circular RNAs are abundantly expressed and upregulated during human epidermal stem cell differentiation. RNA Biol..

[34] Li H., Yang J., Wei X., Song C., Dong D., Huang Y., Lan X., Plath M., Lei C., Ma Y. (2018). CircFUT10 reduces proliferation and facilitates differentiation of myoblasts by sponging miR-133a. J. Cell. Physiol..

[35] Fatica A., Bozzoni I. (2014). Long non-coding RNAs: New players in cell differentiation and development. Nat. Rev. Genet..

[36] Venø M.T., Hansen T.B., Venø S.T., Clausen B.H., Grebing M., Finsen B., Holm I.E., Kjems J. (2015). Spatio-temporal regulation of circular RNA expression during porcine embryonic brain development. Genome Biol..

[37] Greene J., Baird A.-M., Brady L., Lim M., Gray S.G., McDermott R., Finn S.P. (2017). Circular RNAs: Biogenesis, Function and Role in Human Diseases. Front. Mol. Biosci..

[38] Kristensen L.S., Hansen T.B., Venø M.T., Kjems J. (2017). Circular RNAs in cancer: Opportunities and challenges in the field. Oncogene.

[39] Bhan A., Soleimani M., Mandal S.S. (2017). Long Noncoding RNA and Cancer: A New Paradigm. Cancer Res..

[40] Gutschner T., Diederichs S. (2012). The hallmarks of cancer: A long non-coding RNA point of view. RNA Biol..

[41] Küppers R., Klein U., Hansmann M.L., Rajewsky K. (1999). Cellular origin of human B-cell lymphomas. N. Engl. J. Med..

[42] Bonnal R.J.P., Ranzani V., Arrigoni A., Curti S., Panzeri I., Gruarin P., Abrignani S., Rossetti G., Pagani M. (2015). De novo transcriptome profiling of highly purified human lymphocytes primary cells. Sci. Data.

[43] Casero D., Sandoval S., Seet C.S., Scholes J., Zhu Y., Ha V.L., Luong A., Parekh C., Crooks G.M. (2015). Long non-coding RNA profiling of human lymphoid progenitor cells reveals transcriptional divergence of B cell and T cell lineages. Nat. Immunol..

[44] Petri A., Dybkær K., Bøgsted M., Thrue C.A., Hagedorn P.H., Schmitz A., Bødker J.S., Johnsen H.E., Kauppinen S. (2015). Long Noncoding RNA Expression during Human B-Cell Development. PLoS ONE.

[45] Ranzani V., Rossetti G., Panzeri I., Arrigoni A., Bonnal R.J.P., Curti S., Gruarin P., Provasi E., Sugliano E., Marconi M. (2015). LincRNA landscape in human lymphocytes highlights regulation of T cell differentiation by linc-MAF-4. Nat. Immunol..

[46] Tayari M.M., Winkle M., Kortman G., Sietzema J., de Jong D., Terpstra M., Mestdagh P., Kroese F.G.M., Visser L., Diepstra A. (2016). Long Noncoding RNA Expression Profiling in Normal B-Cell Subsets and Hodgkin Lymphoma Reveals Hodgkin and Reed-Sternberg Cell–Specific Long Noncoding RNAs. Am. J. Pathol..

[47] Brazao T.F., Johnson J.S., Muller J., Heger A., Ponting C.P., Tybulewicz V.L. (2016). Long noncoding RNAs in B-cell development and activation. Blood.

[48] Nutt S.L., Heavey B., Rolink A.G., Busslinger M. (1999). Commitment to the B-lymphoid lineage depends on the transcription factor Pax5. Nature.

[49] Verma-Gaur J., Torkamani A., Schaffer L., Head S.R., Schork N.J., Feeney A.J. (2012). Noncoding transcription within the Igh distal VH region at PAIR elements affects the 3D structure of the Igh locus in pro-B cells. Proc. Natl. Acad. Sci. USA.

[50] Liu H., Schmidt-Supprian M., Shi Y., Hobeika E., Barteneva N., Jumaa H., Pelanda R., Reth M., Skok J., Rajewsky K. (2007). Yin Yang 1 is a critical regulator of B-cell development. Genes Dev.

[51] Syrett C.M., Sindhava V., Hodawadekar S., Myles A., Liang G., Zhang Y., Nandi S., Cancro M., Atchison M., Anguera M.C. (2017). Loss of Xist RNA from the inactive X during B cell development is restored in a dynamic YY1-dependent two-step process in activated B cells. PLoS Genet..

[52] DeKoter R.P., Singh H. (2000). Regulation of B Lymphocyte and Macrophage Development by Graded Expression of PU.1. Science.

[53] Rosenbauer F., Owens B.M., Yu L., Tumang J.R., Steidl U., Kutok J.L., Clayton L.K., Wagner K., Scheller M., Iwasaki H. (2006). Lymphoid cell growth and transformation are suppressed by a key regulatory element of the gene encoding PU.1. Nat. Genet..

[54] Mortazavi A., Williams B.A., McCue K., Schaeffer L., Wold B. (2008). Mapping and quantifying mammalian transcriptomes by RNA-Seq. Nat. Methods.

[55] Fang K., Han B.-W., Chen Z.-H., Lin K.-Y., Zeng C.-W., Li X.-J., Li J.-H., Luo X.-Q., Chen Y.-Q. (2014). A distinct set of long non-coding RNAs in childhood *MLL*-rearranged acute lymphoblastic leukemia: Biology and epigenetic target. Hum. Mol. Genet..

[56] Pui C.H., Behm F.G., Downing J.R., Hancock M.L., Shurtleff S.A., Ribeiro R.C., Head D.R., Mahmoud H.H., Sandlund J.T., Furman W.L. (1994). 11q23/MLL rearrangement confers a poor prognosis in infants with acute lymphoblastic leukemia. J. Clin. Oncol..

[57] Fernando T.R., Rodriguez-Malave N.I., Waters E.V., Yan W., Casero D., Basso G., Pigazzi M., Rao D.S. (2015). LncRNA Expression Discriminates Karyotype and Predicts Survival in B-Lymphoblastic Leukemia. Mol. Cancer Res..

[58] Rodríguez-Malavé N.I., Fernando T.R., Patel P.C., Contreras J.R., Palanichamy J.K., Tran T.M., Anguiano J., Davoren M.J., Alberti M.O., Pioli K.T. (2015). BALR-6 regulates cell growth and cell survival in B-lymphoblastic leukemia. Mol. Cancer.

[59] Fernando T.R., Contreras J.R., Zampini M., Rodriguez-Malave N.I., Alberti M.O., Anguiano J., Tran T.M., Palanichamy J.K., Gajeton J., Ung N.M. (2017). The lncRNA CASC15 regulates SOX4 expression in RUNX1-rearranged acute leukemia. Mol. Cancer.

[60] Ghazavi F., De Moerloose B., Van Loocke W., Wallaert A., Helsmoortel H.H., Ferster A., Bakkus M., Plat G., Delabesse E., Uyttebroeck A. (2016). Unique long non-coding RNA expression signature in ETV6/RUNX1-driven B-cell precursor acute lymphoblastic leukemia. Oncotarget.

[61] Moorman A.V., Ensor H.M., Richards S.M., Chilton L., Schwab C., Kinsey S.E., Vora A., Mitchell C.D., Harrison C.J. (2010). Prognostic effect of chromosomal abnormalities in childhood B-cell precursor acute lymphoblastic leukaemia: Results from the UK Medical Research Council ALL97/99 randomised trial. Lancet Oncol..

[62] Lajoie M., Drouin S., Caron M., St-Onge P., Ouimet M., Gioia R., Lafond M.-H., Vidal R., Richer C., Oualkacha K. (2017). Specific expression of novel long non-coding RNAs in high-hyperdiploid childhood acute lymphoblastic leukemia. PLoS ONE.

[63] Wang X., Sehgal L., Jain N., Khashab T., Mathur R., Samaniego F. (2016). LncRNA MALAT1 promotes development of mantle cell lymphoma by associating with EZH2. J. Transl. Med..

[64] Li L.-J., Chai Y., Guo X.-J., Chu S.-L., Zhang L.-S. (2017). The effects of the long non-coding RNA MALAT-1 regulated autophagy-related signaling pathway on chemotherapy resistance in diffuse large B-cell lymphoma. Biomed. Pharmacother..

[65] Ronchetti D., Agnelli L., Taiana E., Galletti S., Manzoni M., Todoerti K., Musto P., Strozzi F., Neri A. (2016). Distinct lncRNA transcriptional fingerprints characterize progressive stages of multiple myeloma. Oncotarget.

[66] Cho S.-F., Chang Y.C., Chang C.-S., Lin S.-F., Liu Y.-C., Hsiao H.-H., Chang J.-G., Liu T.-C. (2014). MALAT1 long non-coding RNA is overexpressed in multiple myeloma and may serve as a marker to predict disease progression. BMC Cancer.

[67] Blume C.J., Hotz-Wagenblatt A., Hüllein J., Sellner L., Jethwa A., Stolz T., Slabicki M., Lee K., Sharathchandra A., Benner A. (2015). p53-dependent non-coding RNA networks in chronic lymphocytic leukemia. Leukemia.

[68] Isin M., Ozgur E., Cetin G., Erten N., Aktan M., Gezer U., Dalay N. (2014). Investigation of circulating lncRNAs in B-cell neoplasms. Clin. Chim. Acta.

[69] Yilan W., Han W. (2017). LncRNA NEAT1 promotes dexamethasone resistance in multiple myeloma by targeting miR-193a/MCL1 pathway. J. Biochem. Mol. Toxicol..

[70] Sattari A., Siddiqui H., Moshiri F., Ngankeu A., Nakamura T., Kipps T.J., Croce C.M. (2016). Upregulation of long noncoding RNA MIAT in aggressive form of chronic lymphocytic leukemias. Oncotarget.

[71] Sone M., Hayashi T., Tarui H., Agata K., Takeichi M., Nakagawa S. (2007). The mRNA-like noncoding RNA Gomafu constitutes a novel nuclear domain in a subset of neurons. J. Cell Sci..

[72] Ronchetti D., Agnelli L., Pietrelli A., Todoerti K., Manzoni M., Taiana E., Neri A. (2018). A compendium of long non-coding RNAs transcriptional fingerprint in multiple myeloma. Sci. Rep..

[73] Subhash S., Andersson P.-O., Kosalai S.T., Kanduri C., Kanduri M. (2016). Global DNA methylation profiling reveals new insights into epigenetically deregulated protein coding and long noncoding RNAs in CLL. Clin. Epigenet..

[74] Khalil A.M., Guttman M., Huarte M., Garber M., Raj A., Rivea Morales D., Thomas K., Presser A., Bernstein B.E., van Oudenaarden A. (2009). Many human large intergenic noncoding RNAs associate with chromatin-modifying complexes and affect gene expression. Proc. Natl. Acad. Sci. USA.

[75] Meng Y.-B., He X., Huang Y.-F., Wu Q.-N., Zhou Y.-C., Hao D.-J. (2017). Long Noncoding RNA CRNDE Promotes Multiple Myeloma Cell Growth by Suppressing miR-451. Oncol. Res. Featur. Preclin. Clin. Cancer Ther..

[76] Gao H.-Y., Wu B., Yan W., Gong Z.-M., Sun Q., Wang H.-H., Yang W. (2017). Microarray expression profiles of long non-coding RNAs in germinal center-like diffuse large B-cell lymphoma. Oncol. Rep..

[77] Zhu D., Fang C., Li X., Geng Y., Li R., Wu C., Jiang J., Wu C. (2017). Predictive analysis of long non-coding RNA expression profiles in diffuse large B-cell lymphoma. Oncotarget.

[78] Yan Y., Han J., Li Z., Yang H., Sui Y., Wang M. (2016). Elevated RNA expression of long non-coding HOTAIR promotes cell proliferation and predicts a poor prognosis in patients with diffuse large B cell lymphoma. Mol. Med. Rep..

[79] Oh E.J., Kim S.H., Yang W.I., Ko Y.H., Yoon S.O. (2016). Long Non-coding RNA HOTAIR Expression in Diffuse Large B-Cell Lymphoma: In Relation to Polycomb Repressive Complex Pathway Proteins and H3K27 Trimethylation. J. Pathol. Transl. Med..

[80] Gupta R.A., Shah N., Wang K.C., Kim J., Horlings H.M., Wong D.J., Tsai M.-C., Hung T., Argani P., Rinn J.L. (2010). Long non-coding RNA HOTAIR reprograms chromatin state to promote cancer metastasis. Nature.

[81] Mourtada-Maarabouni M., Williams G.T. (2014). Role of GAS5 noncoding RNA in mediating the effects of rapamycin and its analogues on mantle cell lymphoma cells. Clin. Lymphoma Myeloma Leuk..

[82] Lenka S., Barbora G., Veronika K., Lenka R., Jana F., Jiri J., Lucie B., Roberta V., Martina A., Miroslav P. (2017). Deregulated expression of long non-coding RNA UCA1 in multiple myeloma. Eur. J. Haematol..

[83] Zhang Z.-S., Wang J., Zhu B.-Q., Ge L. (2018). Long noncoding RNA UCA1 promotes multiple myeloma cell growth by targeting TGF-β. Eur. Rev. Med. Pharmacol. Sci..

[84] Pan Y., Chen H., Shen X., Wang X., Ju S., Lu M., Cong H. (2018). Serum level of long noncoding RNA H19 as a diagnostic biomarker of multiple myeloma. Clin. Chim. Acta.

[85] Sun Y., Pan J., Zhang N., Wei W., Yu S., Ai L. (2017). Knockdown of long non-coding RNA H19 inhibits multiple myeloma cell growth via NF-κB pathway. Sci. Rep..

[86] Ouimet M., Drouin S., Lajoie M., Caron M., St-Onge P., Gioia R., Richer C., Sinnett D. (2017). A childhood acute lymphoblastic leukemia-specific lncRNA implicated in prednisolone resistance, cell proliferation, and migration. Oncotarget.

[87] Arthur G., Almamun M., Taylor K. (2017). Hypermethylation of antisense long noncoding RNAs in acute lymphoblastic leukemia. Epigenomics.

[88] Sánchez Y., Segura V., Marín-Béjar O., Athie A., Marchese F.P., González J., Bujanda L., Guo S., Matheu A., Huarte M. (2014). Genome-wide analysis of the human p53 transcriptional network unveils a lncRNA tumour suppressor signature. Nat. Commun..

[89] Dohner H., Fischer K., Bentz M., Hansen K., Benner A., Cabot G., Diehl D., Schlenk R., Coy J., Stilgenbauer S. (1995). p53 gene deletion predicts for poor survival and non-response to therapy with purine analogs in chronic B-cell leukemias. Blood.

[90] Ouillette P., Collins R., Shakhan S., Li J., Li C., Shedden K., Malek S.N. (2011). The prognostic significance of various 13q14 deletions in chronic lymphocytic leukemia. Clin. Cancer Res..

[91] Garding A., Bhattacharya N., Claus R., Ruppel M., Tschuch C., Filarsky K., Idler I., Zucknick M., Caudron-Herger M., Oakes C. (2013). Epigenetic Upregulation of lncRNAs at 13q14.3 in Leukemia Is Linked to the *In Cis* Downregulation of a Gene Cluster That Targets NF-kB. PLoS Genet..

[92] Wang L.Q., Wong K.Y., Li Z.H., Chim C.S. (2016). Epigenetic silencing of tumor suppressor long non-coding RNA BM742401 in chronic lymphocytic leukemia. Oncotarget.

[93] Ronchetti D., Manzoni M., Agnelli L., Vinci C., Fabris S., Cutrona G., Matis S., Colombo M., Galletti S., Taiana E. (2016). lncRNA profiling in early-stage chronic lymphocytic leukemia identifies transcriptional fingerprints with relevance in clinical outcome. Blood Cancer J..

[94] Ferreira P.G., Jares P., Rico D., Gómez-López G., Martínez-Trillos A., Villamor N., Ecker S., González-Pérez A., Knowles D.G. (2014). Transcriptome characterization by RNA sequencing identifies a major molecular and clinical subdivision in chronic lymphocytic leukemia. Genome Res..

[95] Miller C.R., Ruppert A.S., Fobare S., Chen T.L., Liu C., Lehman A., Blachly J.S., Zhang X., Lucas D.M., Grever M.R. (2017). The long noncoding RNA, treRNA, decreases DNA damage and is associated with poor response to chemotherapy in chronic lymphocytic leukemia. Oncotarget.

[96] Riccardo B., Alejandro R., Tiziana D., Giancarlo C., Tycho B., Julio D., Armando L., Antonella Z., Michele D., Vanessa B. (2018). Expression of the transcribed ultraconserved region 70 and the related long non-coding RNA AC092652.2-202 has prognostic value in Chronic Lymphocytic Leukaemia. Br. J. Haematol..

[97] Mack G.S. (2010). To selectivity and beyond. Nat. Biotechnol..

[98] Miranda T.B., Cortez C.C., Yoo C.B., Liang G., Abe M., Kelly T.K., Marquez V.E., Jones P.A. (2009). DZNep is a global histone methylation inhibitor that reactivates developmental genes not silenced by DNA methylation. Mol. Cancer Ther..

[99] Fiskus W., Rao R., Balusu R., Ganguly S., Tao J., Sotomayor E., Mudunuru U., Smith J.E., Hembruff S.L., Atadja P. (2012). Superior Efficacy of a Combined Epigenetic Therapy against Human Mantle Cell Lymphoma Cells. Clin. Cancer Res..

[100] Oki Y., Buglio D., Fanale M., Fayad L., Copeland A., Romaguera J., Kwak L.W., Pro B., de Castro Faria S., Neelapu S. (2013). Phase I Study of Panobinostat plus Everolimus in Patients with Relapsed or Refractory Lymphoma. Clin. Cancer Res..

[101] Visser H.P.J., Gunster M.J., Kluin-Nelemans H.C., Manders E.M.M., Raaphorst F.M., Meijer C.J.L.M., Willemze R., Otte A.P. (2001). The Polycomb group protein EZH2 is upregulated in proliferating, cultured human mantle cell lymphoma. Br. J. Haematol..

[102] Kienle D., Katzenberger T., Ott G., Saupe D., Benner A., Kohlhammer H., Barth T.F.E., Höller S., Kalla J., Rosenwald A. (2007). Quantitative Gene Expression Deregulation in Mantle-Cell Lymphoma: Correlation with Clinical and Biologic Factors. J. Clin. Oncol..

[103] Hu G., Gupta S.K., Troska T.P., Nair A., Gupta M. (2017). Long non-coding RNA profile in mantle cell lymphoma identifies a functional lncRNA ROR1-AS1 associated with EZH2/PRC2 complex. Oncotarget.

[104] Sehgal L., Mathur R., Braun F.K., Wise J.F., Berkova Z., Neelapu S., Kwak L.W., Samaniego F. (2014). FAS-antisense 1 lncRNA and production of soluble versus membrane Fas in B-cell lymphoma. Leukemia.

[105] Ren D., Li H., Li R., Sun J., Guo P., Han H., Yang Y., Li J. (2016). Novel insight into MALAT-1 in cancer: Therapeutic targets and clinical applications. Oncol. Lett..

[106] Tripathi V., Shen Z., Chakraborty A., Giri S., Freier S.M., Wu X., Zhang Y., Gorospe M., Prasanth S.G., Lal A. (2013). Long Noncoding RNA MALAT1 Controls Cell Cycle Progression by Regulating the Expression of Oncogenic Transcription Factor B-MYB. PLoS Genet..

[107] Verma A., Jiang Y., Du W., Fairchild L., Melnick A., Elemento O. (2015). Transcriptome sequencing reveals thousands of novel long non-coding RNAs in B cell lymphoma. Genome Med..

[108] Sun J., Cheng L., Shi H., Zhang Z., Zhao H., Wang Z., Zhou M. (2016). A potential panel of six-long non-coding RNA signature to improve survival prediction of diffuse large-B-cell lymphoma. Sci. Rep..

[109] Zhou M., Zhao H., Xu W., Bao S., Cheng L., Sun J. (2017). Discovery and validation of immune-associated long non-coding RNA biomarkers associated with clinically molecular subtype and prognosis in diffuse large B cell lymphoma. Mol. Cancer.

[110] Lu Z., Pannunzio N.R., Greisman H.A., Casero D., Parekh C., Lieber M.R. (2015). Convergent BCL6 and lncRNA promoters demarcate the major breakpoint region for BCL6 translocations. Blood.

[111] Wang Y., Zhang M., Xu H., Wang Y., Li Z., Chang Y., Wang X., Fu X., Zhou Z., Yang S. (2017). Discovery and validation of the tumor-suppressive function of long noncoding RNA PANDA in human diffuse large B-cell lymphoma through the inactivation of MAPK/ERK signaling pathway. Oncotarget.

[112] Peng W., Fan H., Wu G., Wu J., Feng J. (2016). Upregulation of long noncoding RNA PEG10 associates with poor prognosis in diffuse large B cell lymphoma with facilitating tumorigenicity. Clin. Exp. Med..

[113] Zhao S., Fang S., Liu Y., Li X., Liao S., Chen J., Liu J., Zhao L., Li H., Zhou W. (2017). The long non-coding RNA NONHSAG026900 predicts prognosis as a favorable biomarker in patients with diffuse large B-cell lymphoma. Oncotarget.

[114] Pan Y., Li H., Guo Y., Luo Y., Li H., Xu Y., Deng J., Sun B. (2016). A pilot study of long noncoding RNA expression profiling by microarray in follicular lymphoma. Gene.

[115] Taub R., Kirsch I., Morton C., Lenoir G., Swan D., Tronick S., Aaronson S., Leder P. (1982). Translocation of the c-myc gene into the immunoglobulin heavy chain locus in human Burkitt lymphoma and murine plasmacytoma cells. Proc. Natl. Acad. Sci. USA.

[116] Dalla-Favera R., Bregni M., Erikson J., Patterson D., Gallo R.C., Croce C.M. (1982). Human c-myc onc gene is located on the region of chromosome 8 that is translocated in Burkitt lymphoma cells. Proc. Natl. Acad. Sci. USA.

[117] Doose G., Haake A., Bernhart S.H., López C., Duggimpudi S., Wojciech F., Bergmann A.K., Borkhardt A., Burkhardt B., Claviez A. (2015). MINCR is a MYC-induced lncRNA able to modulate MYC’s transcriptional network in Burkitt lymphoma cells. Proc. Natl. Acad. Sci. USA.

[118] Winkle M., van den Berg A., Tayari M., Sietzema J., Terpstra M., Kortman G., de Jong D., Visser L., Diepstra A., Kok K. (2015). Long noncoding RNAs as a novel component of the Myc transcriptional network. FASEB J..

[119] Ronchetti D., Manzoni M., Todoerti K., Neri A., Agnelli L. (2016). In Silico Characterization of miRNA and Long Non-Coding RNA Interplay in Multiple Myeloma. Genes.

[120] Zhou M., Zhao H., Wang Z., Cheng L., Yang L., Shi H., Yang H., Sun J. (2015). Identification and validation of potential prognostic lncRNA biomarkers for predicting survival in patients with multiple myeloma. J. Exp. Clin. Cancer Res..

[121] Hu A.-X., Huang Z.-Y., Zhang L., Shen J. (2017). Potential prognostic long non-coding RNA identification and their validation in predicting survival of patients with multiple myeloma. Tumor Biol..

[122] Shen Y., Feng Y., Chen H., Huang L., Wang F., Bai J., Yang Y., Wang J., Zhao W., Jia Y. (2018). Focusing on long non-coding RNA dysregulation in newly diagnosed multiple myeloma. Life Sci..

[123] Samur M.K., Minvielle S., Gulla A., Fulciniti M., Cleynen A., Aktas Samur A., Szalat R., Shammas M., Magrangeas F., Tai Y.-T. (2018). Long intergenic non-coding RNAs have an independent impact on survival in multiple myeloma. Leukemia.

[124] Wenzhuo Z., Xueping G., Sijun Y., Moli H., Wenyue Z., Ping C., Xiaohui Z., Jinxiang F., Jing Q., Bingzong L. (2015). Upregulation of lncRNA MEG3 Promotes Osteogenic Differentiation of Mesenchymal Stem Cells from Multiple Myeloma Patients By Targeting BMP4 Transcription. Stem Cells.

[125] Benetatos L., Dasoula A., Hatzimichael E., Georgiou I., Syrrou M., Bourantas K.L. (2008). Promoter Hypermethylation of the MEG3 (DLK1/MEG3) Imprinted Gene in Multiple Myeloma. Clin. Lymphoma Myeloma.

[126] Wong K.Y., Li Z., Zhang X., Leung G.K.K., Chan G.C.-f., Chim C.S. (2015). Epigenetic silencing of a long non-coding RNA KIAA0495 in multiple myeloma. Mol. Cancer.

[127] Zhan F., Barlogie B., Arzoumanian V., Huang Y., Williams D.R., Hollmig K., Pineda-Roman M., Tricot G., van Rhee F., Zangari M. (2007). Gene-expression signature of benign monoclonal gammopathy evident in multiple myeloma is linked to good prognosis. Blood.

[128] Poi M.J., Li J., Sborov D.W., VanGundy Z., Cho Y.K., Lamprecht M., Pichiorri F., Phelps M.A., Hofmeister C.C. (2017). Polymorphism in ANRIL is associated with relapse in patients with multiple myeloma after autologous stem cell transplant. Mol. Carcinog..

[129] Yang N., Chen J., Zhang H., Wang X., Yao H., Peng Y., Zhang W. (2017). LncRNA OIP5-AS1 loss-induced microRNA-410 accumulation regulates cell proliferation and apoptosis by targeting KLF10 via activating PTEN/PI3K/AKT pathway in multiple myeloma. Cell Death Dis..

[130] Li Q., Chen L., Hu N., Zhao H. (2018). Long non-coding RNA FEZF1-AS1 promotes cell growth in multiple myeloma via miR-610/Akt3 axis. Biomed. Pharmacother..

[131] Chen L., Hu N., Wang C., Zhao H., Gu Y. (2018). Long non-coding RNA CCAT1 promotes multiple myeloma progression by acting as a molecular sponge of miR-181a-5p to modulate HOXA1 expression. Cell Cycle.

[132] Malek E., Kim B., Driscoll J. (2016). Identification of Long Non-Coding RNAs Deregulated in Multiple Myeloma Cells Resistant to Proteasome Inhibitors. Genes.

[133] Brocke-Heidrich K., Kretzschmar A.K., Pfeifer G., Henze C., Löffler D., Koczan D., Thiesen H.-J., Burger R., Gramatzki M., Horn F. (2004). Interleukin-6—Dependent gene expression profiles in multiple myeloma INA-6 cells reveal a Bcl-2 family–independent survival pathway closely associated with Stat3 activation. Blood.

[134] Binder S., Hösler N., Riedel D., Zipfel I., Buschmann T., Kämpf C., Reiche K., Burger R., Gramatzki M., Hackermüller J. (2017). STAT3-induced long noncoding RNAs in multiple myeloma cells display different properties in cancer. Sci. Rep..

[135] Shen X., Zhang Y., Wu X., Guo Y., Shi W., Qi J., Cong H., Wang X., Wu X., Ju S. (2017). Upregulated lncRNA-PCAT1 is closely related to clinical diagnosis of multiple myeloma as a predictive biomarker in serum. Cancer Biomark..

[136] Hansen T.B., Kjems J., Damgaard C.K. (2013). Circular RNA and miR-7 in cancer. Cancer Res..

[137] Hansen T.B. (2018). Improved circRNA Identification by Combining Prediction Algorithms. Front. Cell Dev. Biol..

[138] Szabo L., Salzman J. (2016). Detecting circular RNAs: Bioinformatic and experimental challenges. Nat. Rev. Genet..

[139] Chen D.F., Zhang L.J., Tan K., Jing Q. (2018). Application of droplet digital PCR in quantitative detection of the cell-free circulating circRNAs. Biotechnol. Biotechnol. Equip..

[140] Nicolet B.P., Engels S., Aglialoro F., van den Akker E., von Lindern M.M., Wolkers M.C. (2018). Circular RNA expression in human hematopoietic cells is widespread and cell-type specific. bioRxiv.

[141] Xia P., Wang S., Ye B., Du Y., Li C., Xiong Z., Qu Y., Fan Z. (2018). A Circular RNA Protects Dormant Hematopoietic Stem Cells from DNA Sensor cGAS-Mediated Exhaustion. Immunity.

[142] Guarnerio J., Bezzi M., Jeong J.C., Paffenholz S.V., Berry K., Naldini M.M., Lo-Coco F., Tay Y., Beck A.H., Pandolfi P.P. (2016). Oncogenic Role of Fusion-circRNAs Derived from Cancer-Associated Chromosomal Translocations. Cell.

[143] Dzikiewicz-Krawczyk A., Kok K., Slezak-Prochazka I., Robertus J.-L., Bruining J., Tayari M.M., Rutgers B., de Jong D., Koerts J., Seitz A. (2017). ZDHHC11 and ZDHHC11B are critical novel components of the oncogenic MYC-miR-150-MYB network in Burkitt lymphoma. Leukemia.

[144] Yang Q., Du W.W., Wu N., Yang W., Awan F.M., Fang L., Ma J., Li X., Zeng Y., Yang Z. (2017). A circular RNA promotes tumorigenesis by inducing c-myc nuclear translocation. Cell Death Differ..

[145] Dahl M., Daugaard I., Andersen M., Hansen T.B., Grønbæk K., Kjems J., Kristensen L.S. (2018). Enzyme-free digital counting of endogenous circular RNA molecules in B-cell malignancies. Lab. Investig..

[146] Weng W., Wei Q., Toden S., Yoshida K., Nagasaka T., Fujiwara T., Cai S., Qin H., Ma Y., Goel A. (2017). Circular RNA ciRS-7—A Promising Prognostic Biomarker and a Potential Therapeutic Target in Colorectal Cancer. Clin. Cancer Res..

[147] Barbagallo D., Condorelli A., Ragusa M., Salito L., Sammito M., Banelli B., Caltabiano R., Barbagallo G., Zappalà A., Battaglia R. (2016). Dysregulated miR-671-5p / CDR1-AS / CDR1 / VSNL1 axis is involved in glioblastoma multiforme. Oncotarget.

[148] Zheng Q., Bao C., Guo W., Li S., Chen J., Chen B., Luo Y., Lyu D., Li Y., Shi G. (2016). Circular RNA profiling reveals an abundant circHIPK3 that regulates cell growth by sponging multiple miRNAs. Nat. Commun..

[149] Okholm T.L.H., Nielsen M.M., Hamilton M.P., Christensen L.-L., Vang S., Hedegaard J., Hansen T.B., Kjems J., Dyrskjøt L., Pedersen J.S. (2017). Circular RNA expression is abundant and correlated to aggressiveness in early-stage bladder cancer. NPJ Genom. Med..

[150] Hsiao K.Y., Lin Y.C., Gupta S.K., Chang N., Yen L., Sun H.S., Tsai S.J. (2017). Noncoding effects of circular RNA CCDC66 promote colon cancer growth and metastasis. Cancer Res..

[151] Barbagallo D., Caponnetto A., Cirnigliaro M., Brex D., Barbagallo C., D’Angeli F., Morrone A., Caltabiano R., Barbagallo G.M., Ragusa M. (2018). CircSMARCA5 Inhibits Migration of Glioblastoma Multiforme Cells by Regulating a Molecular Axis Involving Splicing Factors SRSF1/SRSF3/PTB. Int. J. Mol. Sci..

[152] Yu J., Xu Q., Wang Z., Yang Y., Zhang L., Ma J., Sun S., Yang F., Zhou W. (2018). Circular RNA cSMARCA5 inhibits growth and metastasis in hepatocellular carcinoma. J. Hepatol..

[153] Yao Z., Luo J., Hu K., Lin J., Huang H., Wang Q., Zhang P., Xiong Z., He C., Huang Z. (2017). *ZKSCAN1* gene and its related circular RNA (circ *ZKSCAN1*) both inhibit hepatocellular carcinoma cell growth, migration, and invasion but through different signaling pathways. Mol. Oncol..

[154] Craig V.J., Cogliatti S.B., Imig J., Renner C., Neuenschwander S., Rehrauer H., Schlapbach R., Dirnhofer S., Tzankov A., Müller A. (2011). Myc-mediated repression of microRNA-34a promotes high-grade transformation of B-cell lymphoma by dysregulation of FoxP1. Blood.

[155] Chen Z., Yan C.T., Dou Y., Viboolsittiseri S.S., Wang J.H. (2013). The role of a newly identified SET domain-containing protein, SETD3, in oncogenesis. Haematologica.

[156] Morin R.D., Johnson N.A., Severson T.M., Mungall A.J., An J., Goya R., Paul J.E., Boyle M., Woolcock B.W., Kuchenbauer F. (2010). Somatic mutations altering EZH2 (Tyr641) in follicular and diffuse large B-cell lymphomas of germinal-center origin. Nat. Genet..

[157] Stilgenbauer S., Winkler D., Ott G., Schaffner C., Leupolt E., Bentz M., Möller P., Müller-Hermelink H.K., James M.R., Lichter P. (1999). Molecular characterization of 11q deletions points to a pathogenic role of the ATM gene in mantle cell lymphoma. Blood.

[158] Camus V., Miloudi H., Taly A., Sola B., Jardin F. (2017). XPO1 in B cell hematological malignancies: From recurrent somatic mutations to targeted therapy. J. Hematol. Oncol..

[159] Ahsmann E.J., Lokhorst H.M., Dekker A.W., Bloem A.C. (1992). Lymphocyte function-associated antigen-1 expression on plasma cells correlates with tumor growth in multiple myeloma. Blood.

[160] Chesi M., Nardini E., Lim R.S., Smith K.D., Kuehl W.M., Bergsagel P.L. (1998). The t(4;14) translocation in myeloma dysregulates both FGFR3 and a novel gene, MMSET, resulting in IgH/MMSET hybrid transcripts. Blood.

[161] Lu G., Middleton R.E., Sun H., Naniong M.V., Ott C.J., Mitsiades C.S., Wong K.K., Bradner J.E., Kaelin W.G. (2014). The myeloma drug lenalidomide promotes the cereblon-dependent destruction of ikaros proteins. Science.

[162] Glazar P., Papavasileiou P., Rajewsky N. (2014). circBase: A database for circular RNAs. RNA.

[163] Geiss G.K., Bumgarner R.E., Birditt B., Dahl T., Dowidar N., Dunaway D.L., Fell H.P., Ferree S., George R.D., Grogan T. (2008). Direct multiplexed measurement of gene expression with color-coded probe pairs. Nat. Biotechnol..

[164] Reis P.P., Waldron L., Goswami R.S., Xu W., Xuan Y., Perez-Ordonez B., Gullane P., Irish J., Jurisica I., Kamel-Reid S. (2011). mRNA transcript quantification in archival samples using multiplexed, color-coded probes. BMC Biotechnol..

[165] Aird D., Ross M.G., Chen W.-S., Danielsson M., Fennell T., Russ C., Jaffe D.B., Nusbaum C., Gnirke A. (2011). Analyzing and minimizing PCR amplification bias in Illumina sequencing libraries. Genome Biol..

[166] Kane M.D., Jatkoe T.A., Stumpf C.R., Lu J., Thomas J.D., Madore S.J. (2000). Assessment of the sensitivity and specificity of oligonucleotide (50 mer) microarrays. Nucleic Acids Res..

[167] Ulitsky I. (2016). Evolution to the rescue: Using comparative genomics to understand long non-coding RNAs. Nat. Rev. Genet..

[168] Lennox K.A., Behlke M.A. (2016). Cellular localization of long non-coding RNAs affects silencing by RNAi more than by antisense oligonucleotides. Nucleic Acids Res..

[169] Gutschner T., Baas M., Diederichs S. (2011). Noncoding RNA gene silencing through genomic integration of RNA destabilizing elements using zinc finger nucleases. Genome Res..

[170] Mali P., Yang L., Esvelt K.M., Aach J., Guell M., DiCarlo J.E., Norville J.E., Church G.M. (2013). RNA-Guided Human Genome Engineering via Cas9. Science.

[171] Goyal A., Myacheva K., Groß M., Klingenberg M., Duran Arqué B., Diederichs S. (2017). Challenges of CRISPR/Cas9 applications for long non-coding RNA genes. Nucleic Acids Res..

[172] Denzler R., McGeary S.E., Title A.C., Agarwal V., Bartel D.P., Stoffel M. (2016). Impact of MicroRNA Levels, Target-Site Complementarity, and Cooperativity on Competing Endogenous RNA-Regulated Gene Expression. Mol. Cell.

[173] Jens M., Rajewsky N. (2014). Competition between target sites of regulators shapes post-transcriptional gene regulation. Nat. Rev. Genet..

[174] Gardini A. (2017). Global Run-On sequencing (GRO-seq). Methods Mol. Biol..

[175] Dudekula D.B., Panda A.C., Grammatikakis I., De S., Abdelmohsen K., Gorospe M. (2016). CircInteractome: A web tool for exploring circular RNAs and their interacting proteins and microRNAs. RNA Biol..

[176] Zeng W., Jiang S., Kong X., El-Ali N., Ball Alexander R. J., Ma C.I.-H., Hashimoto N., Yokomori K., Mortazavi A. (2016). Single-nucleus RNA-seq of differentiating human myoblasts reveals the extent of fate heterogeneity. Nucleic Acids Res..

